# The importance of embryology for parents of children with congenital hand differences

**DOI:** 10.1177/17531934211064185

**Published:** 2021-12-08

**Authors:** Andrew D. Clelland, Órla Duncan, Wee L. Lam

**Affiliations:** 1 Edinburgh Medical School, University of Edinburgh, Edinburgh, UK; 2 Department of Plastic and Reconstructive Surgery, Royal Hospital for Sick Children, Edinburgh, UK; 3 Department of Plastic and Reconstructive Surgery, St. John’s Hospital, Livingstone, UK

**Keywords:** Congenital hand difference, embryology, consultation, developmental biology

## Abstract

This study aimed to determine whether embryology knowledge or explaining the possible developmental pathway error was important for parents of affected children, and to secondarily determine if there was a relationship between desired knowledge of embryology and disease severity, maternal age group or maternal level of education. Using a self-administered questionnaire, a significant proportion of responding parents considered knowledge of embryology important (32 out of 43). We found a significant association between the importance of embryology knowledge for parents and the disease severity. However, the importance and level of knowledge desired was not related to maternal age or level of education. This study demonstrated the importance of explaining the associated developmental errors in the congenital hand consultation, particularly in severe anomalies. Surgeons should familiarize themselves with embryology to provide an explanation as to why congenital hand differences happen, which may provide better psychological support for parents of these children.

## Introduction

Explaining about aetiology of a congenital hand difference (CHD) during a congenital hand surgery consultation remains challenging; as the majority do not have a teratogenic, syndromic or known genetic cause. Often it is left to the surgeon to explain the possible developmental errors that have occurred during gestation. Such an explanation requires some description of the normal embryological development of the upper limb, as well as theories of what could have gone wrong. There may be a presumption that parents would not understand information of this complexity, nor be interested in knowing the intricacies of why a CHD happened in their child, precluding the surgeon from going into too many details about path-embryology.

In 2013, the International Federation of Societies for Surgery of the Hand (IFSSH) officially recommended the Oberg, Manske and Tonkin (OMT) system (Goldfarb et al., 2020) to replace the previous Swanson classification, which was an eclectic mixture of dysmorphology and aetiology. This represented a shift to a system based almost entirely on aetiology and developmental biology. Centred around the three axes of upper limb development, the OMT system classifies conditions according to what could go wrong during formation (malformations), growth (deformation and dysplasias) (Tonkin et al., 2013, 2015) or if there is a pre-existing recognized genetic mutation (syndromes). Despite the official adoption of the OMT system by the IFSSH, its uptake remains limited (Lowry et al., 2017). In our experience and from personal communications, one possible explanation may be the reluctance by surgeons to learn embryology in enough detail to use it practically.

This prospective study tries to determine the importance of knowledge of embryology for parents of children born with CHD. A second objective sought to investigate whether there is an association between CHD severity, maternal age, maternal level of education and the desire to obtain more information about embryology.

## Methods

Ethical guidance was obtained from the relevant research ethics service. The Standards for Quality Improvement Reporting Excellence (SQUIRE) study 2.0 reporting guidelines (Ogrinc et al., 2016) were consulted to develop a self-administered questionnaire to measure the importance of embryology of CHDs for parents within a tertiary congenital hand clinic (Online Figure S1). The questionnaire was produced after review of the literature with a multidisciplinary team input, including a consultant congenital hand surgeon of Level 4 experience (Tang and Giddins, 2016) and a nurse specialist in the psychosocial aspects of CHDs. It was then piloted among four families for constructive feedback to improve construct validity and ensure sensitive means of questioning. At this stage, minor alterations were made to the language of the questionnaire to make them more parent-friendly and sensitive. Finally, a specialist patient support charity for upper limb anomalies (REACH, https://reach.org.uk/) was consulted, with no further amendments suggested.. The final questionnaire collected demographic information, including maternal age group at the time of birth, ethnicity, level of education, parity and family history of hand conditions. It enquired about previous information and resources accessed by parents from the local health board and patient-support charities, including genetic consultations. A five-point numerical scale was used to assess parental attitudes to embryology knowledge whereby 1 = no knowledge/not important at all, and 5 = highly important. A further five-point scale was used to grade the level of desired knowledge and likelihood of utilizing an educational resource if provided, as well as resource format. A section comprising 17 elements, including categorical and numerical scale style questions, explored whether parents had encountered the term ‘embryology’ in their own research and how important it was to know why their child’s CHD has occurred. Parents were also asked what additional information they would like and what form these resources should take to make them most helpful. The questionnaire used can be found in the supplementary Online Material (Online Figure S1).

### Data collection

Data was collected from the parents of consecutive patients who presented to the congenital hand clinic over an 11-month period between June 2018 and April 2019 and were willing to participate. Eligible parents were those whose biological child presented with a CHD, whereas exclusion criteria included CHDs attributed to known antenatal risk factors, including foetal distress, pharmacological agents used during pregnancy, such as thalidomide or anticonvulsants, or any history of trauma during pregnancy. It was explained that participation would not affect patient care and was entirely voluntary. Non-biological parents and CHDs, where the aetiology remains controversial, such as trigger digits, were also excluded from the study (Goldfarb et al., 2020). All parents were given an initial brief explanation of normal embryology of the hand with pictorial aids by the consultant congenital hand surgeon, and then the current understanding of developmental errors responsible for the CHD, as guided by the OMT. A summary of the terms used for explaining the aetiology of more common conditions and their known aetiologies, as well as the pictorial aids that were used, are as shown in Online Table S1 and Online Figures S2 and S3.

Verbally consenting parents were then taken to a quiet clinic room to complete the questionnaire, where they were encouraged to provide answers based on their own interpretation of the questions. Severity scores were assigned according to Lam et al.’s (2021) CHD severity classification system (Table 1).

**Table 1.** CHD Severity classification system and proportions of CHD disease severity.

Category	Severity score	Example	ƒ
1	Treatment possible to normal	Simple polydactyly	3
2	Treatment possible to near normal	Simple syndactyly	18
3	Treatment possible but always some hand difference	Symbrachydactyly	20
4	Treatment not possible	Proximal transverse arrest	2

*f*: frequency; CHD: congenital hand difference.

### Statistical methods

Data pertaining to importance of embryology reported on a 1–5 scale was dichotomized, with ratings of 1–3 denoted ‘not important’ (G1) and 4–5 considered ‘important’ (G2) to facilitate a binomial test. Significance was determined if *p* < 0.05 (two-sided). A Fisher’s exact test for small groups (where *n* < 5) of nominal variables was carried out to test the null hypothesis that the relative importance of embryology was independent of disease severity, maternal age and maternal level of education.

## Results

**Figure 1.**
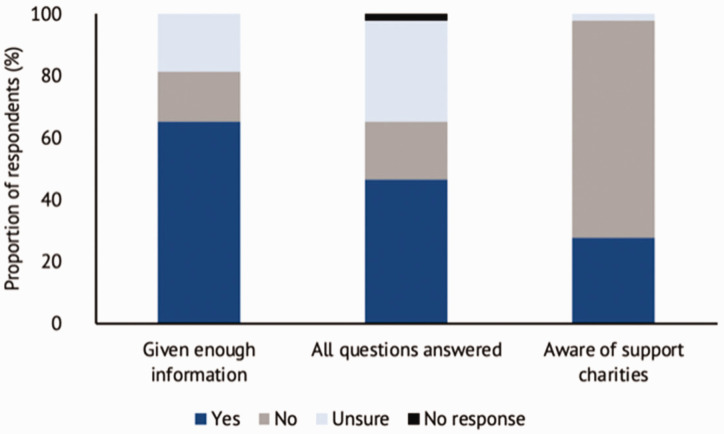
Parental views of existing information for CHDs. CHD: congenital hand difference.

A total of 45 questionnaires were collated for analysis, with two incomplete responses excluded from analysis. Participant demographics (Table 2) and compositions of CHD severity groups are shown in Table 1. Twenty-eight out of the 43 respondents believed they were provided enough information on their child’s CHD, although only 20 of these 28 felt that all of their questions were answered around the time they were told their child had a CHD by the neonatologists or paediatricians (usually the first doctor to communicate with the parents) (Figure 1). Only 12 respondents were aware of CHD support charities, such as REACH (https://reach.org.uk/) and Kidscape (https://www.kidscape.org.uk/) (Figure 1).

**Table 2.** Participant demographic summary.

Demographic	ƒ	Relative ƒ (%)	Cumulative percentile (%)
Maternal age group (years)			
16–19	2	4.6	4.6
20–24	5	11.6	16.2
25–29	15	34.9	51.1
30–34	8	18.6	69.7
35–39	10	23.2	93
40–44	3	7	100
Maternal level of education			
No qualifications	1	2.3	2.3
High school qualifications	9	20.9	23.2
Further education (college degree)	13	30.2	53.4
Higher education (university degree)	20	46.5	100
Family history of CHDs^a^			
Yes	34	81	81
No	8	19	100

a One participant did not provide response.

*f*: frequency; CHD: congenital hand difference.

### Importance of embryology for parents

**Figure 2.**
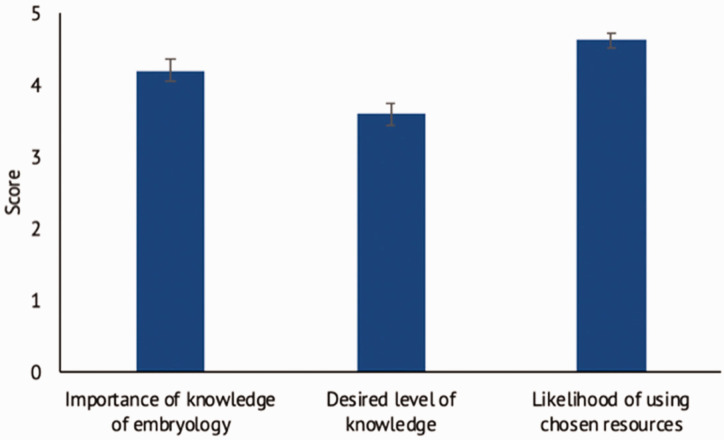
Level of importance of embryology for parents (error bars indicate standard errors).

When assessing the importance of embryology, parents rated a mean of 4.2 (SEM 0.16) (Figure 2). In addition, the observed proportion of G2 (score of 4–5) responses (0.74) was greater than the proportion of G1 (1–3) responses (0.26) (*p* = 0.0019), indicating that embryology was considered important for parents.

When assessing the level of knowledge desired on a five-point scale, parents rated a high level (mean 3.6, SEM 0.16) and indicated they were likely to use their chosen resource 4.6 (SEM 0.1) (Figure 2). The most popular format for future educational resources was via a trusted website (29), followed by one-to-one explanation (26), then leaflet (25) and then video (19).

### Correlations with independent variables

**Figure 3.**
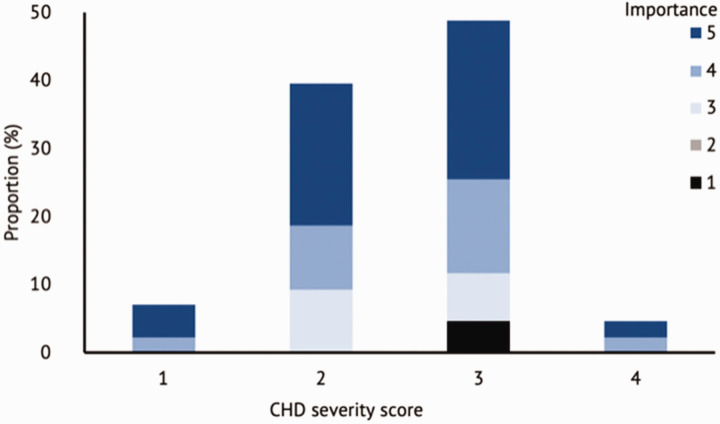
Stacked bar charts of importance of embryology versus disease severity as per Table 1.

There was association between importance of embryology and disease severity (*p* = 0.0323) (Figure 3). Importance of embryology was not associated with maternal age (*p* = 0.7686) or maternal level of education (*p* = 0.4032). There was a non-significant positive trend between increasing maternal level of education and importance of embryology (Online Figures S4 and S5).

## Discussion

Overall, parents desired a high level of embryological knowledge, with more than three-quarters considering knowing ‘why things happen’ to be as important as ‘what would happen next’. Thus, parents are supportive of further counselling on CHD aetiology by consultation and development of supplementary educational resources, such as a trusted website or leaflet (Online Figure S3). This study also demonstrated profound inconsistencies in the information that parents receive at the time of diagnosis and thereafter, with regards to the possible aetiology of their child’s CHD. It was evident that CHD psychosocial support charities are often overlooked, despite knowledge that hand appearance differences may have profoundly detrimental effects on a growing child’s well-being causing stress and anxiety (Franzblau et al., 2015), particularly at stages of development such as starting school. Despite the input of paediatric hand surgeons and therapists, the role of these charities is important in connecting parents to other supportive channels.

Bradbury (2007) has previously observed that the decision-making process for parents, when considering surgery for their child, was more duly influenced by the method of communication of the clinician than the perceived technicalities of the proposed operation. Clinicians were perceived to have the responsibility of communicating the severity and prognosis of a child’s CHD in a parent-friendly, accurate and understandable way (Bradbury, 2007). Surely for a condition like CHD, basic information must include a possible explanation of why things happen. Anecdotally, surgeons are often ill-equipped to explain embryology in a logical, concise and patient-friendly way.

In our clinic, the senior author used visual aids (Online Figure S2) to explain the possible reasons why malformations happened. There are several aetiologies and nuances of developmental biology that cannot be explained, but even so, many parents appreciated the time taken by the surgeon to try and explain what could have gone wrong in the stages of development. The usefulness of the OMT system was clearly evident in explaining the aetiology of CHDs to parents. The fact that it is also regularly updated provides a measure of reassurance that the information is up to date and peer-reviewed (Lam et al., 2020).

We found association between CHD severity and importance of embryology (*p* = 0.0323). This suggests that clinicians should place greater emphasis on explaining upper limb embryology to parents whose child present with more severe CHDs. This differs from a previous review (Benbassat et al., 1998), which reported clinicians may only know how much information to give after direct enquiry of the patient, and in many cases those with more severe disease tended to assume a more passive role. While the binomial test showed that embryology was important for all patients, the importance of embryology was not associated with either maternal age (*p* = 0.7686) or level of education (*p* = 0.4032). The latter finding contrasts with evidence suggesting a link between functional health literacy and level of education (Adams, 2010), however there is insufficient evidence of this link in parents of paediatric surgical patients.

For other congenital conditions, previous publications studied the importance of knowledge of embryology. Within the plastic surgical subspeciality of craniofacial surgery, Crocker and Crocker (1970) noted that even highly educated parents may harbour incorrect ideas about the aetiology of cleft lip and palate, which may imply personal fault and feelings of guilt. There were several instances during the survey that parents remarked this was the first time anyone had ever tried to explain why the CHD happened, and how much they appreciated it, even when a clear explanation could not be given. At the very least, it provided solace when efforts were made to explain what is currently known, and what remains unknown. Furthermore, it allows the parents to ask questions and to leave the consultation with the knowledge that the scientific community is still actively pursuing knowledge that may help other children in the future. It has been shown that educating and empowering patients and their carers improves patient health literacy leading to improved outcomes (Paterick et al., 2017). This early explanation of aetiology may equip parents with the necessary information to talk to their children when their own curiosity about their hand becomes apparent. Patient-reported outcome measures (PROMs) within the field of CHDs should be established in the future, perhaps questions pertaining to how much embryological or causative information they have been given, should be incorporated in the design of validated questionnaires.

A degree of selection bias may have influenced the results of our study. The inherent subjectivity of a questionnaire is an important limitation, as this may confer result bias as responses depend on a respondent’s perception of each question. Given that questions related to the care of their biological child, there is a degree of acquiescence bias. The questionnaire attempted to minimize this effect through use of a five-point numerical scale when assessing parental attitudes. To improve the construct validity of the study, the questionnaire comprised a mix of categorical, scalar and open questions. Another limitation pertains to the measure of disease severity; in the absence of any universal scoring system for the wide heterogeneity of CHDs, we used a previously designed simple scale (Lam et al., 2021) that had shown good parent–surgeon agreement. Large multicentre studies in the future may allow powered analysis between or within severity groups, using established systems to correlate results to determine, for example, the embryology knowledge that the parent of a child with a Blauth 2 thumb may desire, versus that of a child with a Blauth 5 thumb.

Our study has led us to provide parents with additional resources during the consultation (Online Figures S2 and S3), which might be helpful to colleagues to utilize embryology to explain CHD aetiology to parents. Surgeons should familiarize themselves with embryology to provide an explanation as to why CHDs happen, which may provide better psychological support for parents of these children during the consultation process.

## Supplemental Material

sj-pdf-1-jhs-10.1177_17531934211064185 - Supplemental material for The importance of embryology for parents of children with congenital hand differences Click here for additional data file. Supplemental material, sj-pdf-1-jhs-10.1177_17531934211064185 for The importance of embryology for parents of children with congenital hand differences by Andrew D. Clelland, Órla Duncan and Wee L. Lam in Journal of Hand Surgery (European Volume)

sj-pdf-2-jhs-10.1177_17531934211064185 - Supplemental material for The importance of embryology for parents of children with congenital hand differences Click here for additional data file. Supplemental material, sj-pdf-2-jhs-10.1177_17531934211064185 for The importance of embryology for parents of children with congenital hand differences by Andrew D. Clelland, Órla Duncan and Wee L. Lam in Journal of Hand Surgery (European Volume)

sj-pdf-3-jhs-10.1177_17531934211064185 - Supplemental material for The importance of embryology for parents of children with congenital hand differences Click here for additional data file. Supplemental material, sj-pdf-3-jhs-10.1177_17531934211064185 for The importance of embryology for parents of children with congenital hand differences by Andrew D. Clelland, Órla Duncan and Wee L. Lam in Journal of Hand Surgery (European Volume)

sj-pdf-4-jhs-10.1177_17531934211064185 - Supplemental material for The importance of embryology for parents of children with congenital hand differences Click here for additional data file. Supplemental material, sj-pdf-4-jhs-10.1177_17531934211064185 for The importance of embryology for parents of children with congenital hand differences by Andrew D. Clelland, Órla Duncan and Wee L. Lam in Journal of Hand Surgery (European Volume)

sj-pdf-5-jhs-10.1177_17531934211064185 - Supplemental material for The importance of embryology for parents of children with congenital hand differences Click here for additional data file. Supplemental material, sj-pdf-5-jhs-10.1177_17531934211064185 for The importance of embryology for parents of children with congenital hand differences by Andrew D. Clelland, Órla Duncan and Wee L. Lam in Journal of Hand Surgery (European Volume)

sj-pdf-6-jhs-10.1177_17531934211064185 - Supplemental material for The importance of embryology for parents of children with congenital hand differences Click here for additional data file. Supplemental material, sj-pdf-6-jhs-10.1177_17531934211064185 for The importance of embryology for parents of children with congenital hand differences by Andrew D. Clelland, Órla Duncan and Wee L. Lam in Journal of Hand Surgery (European Volume)
