# Supplementary material for: The importance of embryology for parents of children with congenital hand differences
Source: J Hand Surg Eur Vol. 2021 Dec 8;47(5):475–80. doi: 10.1177/17531934211064185 (PMC9008554; doi:10.1177/17531934211064185)
Supplement: sj-pdf-1-jhs-10.1177_17531934211064185 - Supplemental material for The importance of embryology for parents of children with congenital hand differences [file sj-pdf-1-jhs-10.1177_17531934211064185.pdf]

Department of Hand Surgery, Royal Hospital for Sick Children, Edinburgh Service Evaluation Questionnaire:

Dear Parent(s),

As a team we are always looking at ways in which we can improve the service we provide. We invite you to complete this questionnaire which has been designed to look at the level of support we provide to parents with children born with congenital hand differences.

We are trying to understand what knowledge parents currently have of their child's hand difference, and what parents would like to know more about, in terms of why hand differences happen. We hope this will allow us to develop more resources to improve the service for children and families.

Please be assured that information provided will be handled with the strictest confidence. Furthermore, there is no obligation to complete the questionnaire.

If you have any questions regarding this letter, or the questionnaire, please email Andrew Clelland at s1791785@ed.ac.uk.

Yours sincerely,

Andrew Clelland and the Hand Reconstruction Team

This questionnaire should take approx. 10 minutes to complete. Please CIRCLE your chosen response.

*Demographic information will be held in the strictest confidence and reported only as combined characteristics and not as individual data. Demographic information will not be used for any other purpose.

SECTION A: DEMOGRAPGHICS*

**A.**	**Are you a biological parent of the child?**					Yes	No
**B.**	**Is English your first language?**					Yes	No
**C.**	**Age group of mother at time of birth**	16-19	20-24	25-29	30-34	35-39	40-44
**D.**	**Ethnicity**	White	Mixed or multiple ethnic group	Asian	African	Caribbean or Black	Other (please specify)

**E.**	**Level of education**	No qualifications	High school qualifications	Further education (College HNC/HND)	Higher Education (University degree)
**F.**	**Number of children**				
**G.**	**Family medical history of hand conditions?** If *Yes*, please provide some more information:			Yes	No

SECTION B

**1.**	Have you seen a geneticist? If so, what were you told?	Yes	No	Unsure		
**2.**	Do you believe you have been given enough information to support your understanding of your child's hand difference?	Yes	No	Unsure		
**3.**	Do you think the resources available at present answer all of your questions?	Yes	No	Unsure		
**4.**	Have you ever thought about the reasons for your child's hand difference?<br>If *Yes*, could you tell us a little more:	Yes	No	Unsure		
**5.**	Have you heard of hand difference support charities such as REACH, Changing Faces or Kidscape?	Yes	No	Unsure		
**6.**	If so, how useful did you find this information? *(5 = very useful; 1 = not useful at all)*	1	2	3	4	5

SECTION C

**1.**	Were you aware of congenital hand differences before the birth of your child?	Yes	No	Unsure
**2.**	Were the possible causes of your child's hand difference discussed with you at time of diagnosis?	Yes	No	Unsure
**3.**	Have possible causes been discussed with you since then?<br>If *Yes*, could you tell us a little more:	Yes	No	Unsure
**4.**	What do you know about how congenital hand differences happen?			
**5.**	What information would you like about possible causes of congenital hand differences?			

**6.**	How important is it for you to understand how congenital hand differences happen? *(5 = very important; 1 = not important at all)*	1	2	3	4	5
**7.**	Have you previously researched the causes of congenital hand differences?	Yes	No	Unsure		
**8.**	If so, have you come across the term 'embryology?'	Yes	No	Unsure		
**9.**	'Embryology' is the study of how the baby develops in the womb. Do you think that understanding the development of the hand could help parents understand more about the causes of congenital hand differences?	Yes	No	Unsure		
**10.**	Do you think that this would improve your understanding of the reasons for certain treatments?	Yes	No	Unsure		
**11.**	How important do you think that knowledge of embryology is in understanding your child's congenital hand difference? *(5 = very important; 1 = not important at all)*	1	2	3	4	5
**12.**	What level of knowledge would you be comfortable with? *(5 = medical textbook; 1 = no knowledge)*	1	2	3	4	5
**13.**	Do you think that knowing how the hand is formed would change the way you think about hand differences?	Yes	No	Unsure		
**14.**	Do you think it would be helpful to understand the classification system used by the medical team to describe your child's hand difference?	Yes	No	Unsure		
**15.**	What kind of resources would you find useful in understanding embryology? ***(circle all that apply)*** **Leaflet – Website – Video – One to one explanation – Other (please specify) ___________**					
**16.**	How likely are you to use your chosen resource if it were available? *(5 = very likely; 1 = not likely at all)*	1	2	3	4	5
**17.**	What is the single most important piece of information you would like to know about your child's hand difference?					

SECTION D

Please rank the following from 1 to 5 in order of importance (1 = most important; 5 = least important). **Please use each number once only.**

'*I want to know why congenital hand differences happen…*'

	**Reason**	**Rank**
**A.**	To better understand the available treatments	
**B.**	To find out if future pregnancies will be affected	
**C.**	To explain the condition to your child when they are older	
**D.**	To find out if having a hand difference means other parts of the body are affected	
**E.**	For my own peace of mind	

Please share any additional comments you may have:

Thank you for completing this questionnaire.
