# Supplementary material for: The importance of embryology for parents of children with congenital hand differences
Source: J Hand Surg Eur Vol. 2021 Dec 8;47(5):475–80. doi: 10.1177/17531934211064185 (PMC9008554; doi:10.1177/17531934211064185)
Supplement: sj-pdf-2-jhs-10.1177_17531934211064185 - Supplemental material for The importance of embryology for parents of children with congenital hand differences [file sj-pdf-2-jhs-10.1177_17531934211064185.pdf]

Supplementary Table S1

**Conditions**	**OMT classification**	**Phrases used in Explanation of Aetiology**	**References**
**Radial longitudinal deficiency**	Malformation; entire upper limb; radial-ulnar (anterior posterior) axis	"The anterior posterior axis is controlled by the zone of polarizing activity via a responsible protein, the sonic hedgehog SHH). It is thought that there is an error in the regulation of the SHH pathway which leads to suppression of radial structure formation. Thus, the radius and its associated structures did not form."	Guéro S. Developmental biology of the upper limb. Hand Surg Rehabil. 2018 Oct;37(5):265-274
**Transverse arrest**	Malformation; entire upper limb; proximo-distal axis	"The proximal-distal axis is controlled by the apical ectodermal ridge. It is thought that there has been an insult to the ridge during development, which leads to suppression of growth. Thus, the forearm (or hand) did not form."	Saunders JW. The proximo-distal sequence of origin of the parts of the chick wing and the role of the ectoderm. J Exp Zool, 108 (1948), pp. 363-403
**Syndactyly**	Malformation; hand plate; unspecified axis	"Following formation of the digits, there is a process called apoptosis (or programmed cell death) that allows digit separation. The process is tightly controlled by proteins such as bone morphogenetic proteins (BMPs). The exact mechanism remains unknown, but due to errors during development, the fingers fail to separate. Thus, there is resultant syndactyly.'	Malik S. Syndactyly: phenotypes, genetics and current classification. Eur J Hum Genet. 2012 Aug; 20(8): 817–824.
