# Supplementary figures and images for: The importance of embryology for parents of children with congenital hand differences
Source: J Hand Surg Eur Vol. 2021 Dec 8;47(5):475–80. doi: 10.1177/17531934211064185 (PMC9008554; doi:10.1177/17531934211064185)

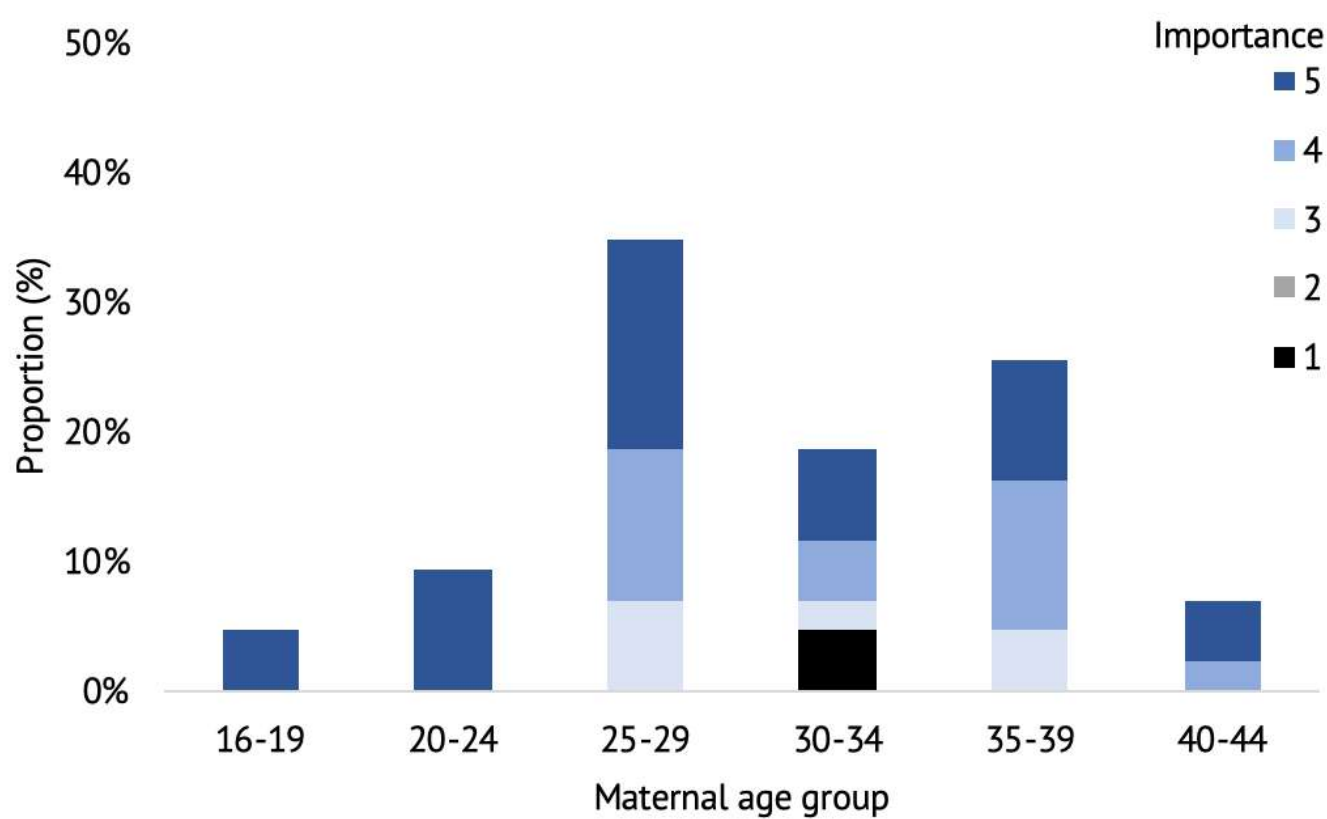

Supplement: sj-pdf-3-jhs-10.1177_17531934211064185 - Supplemental material for The importance of embryology for parents of children with congenital hand differences [file sj-pdf-3-jhs-10.1177_17531934211064185.pdf]

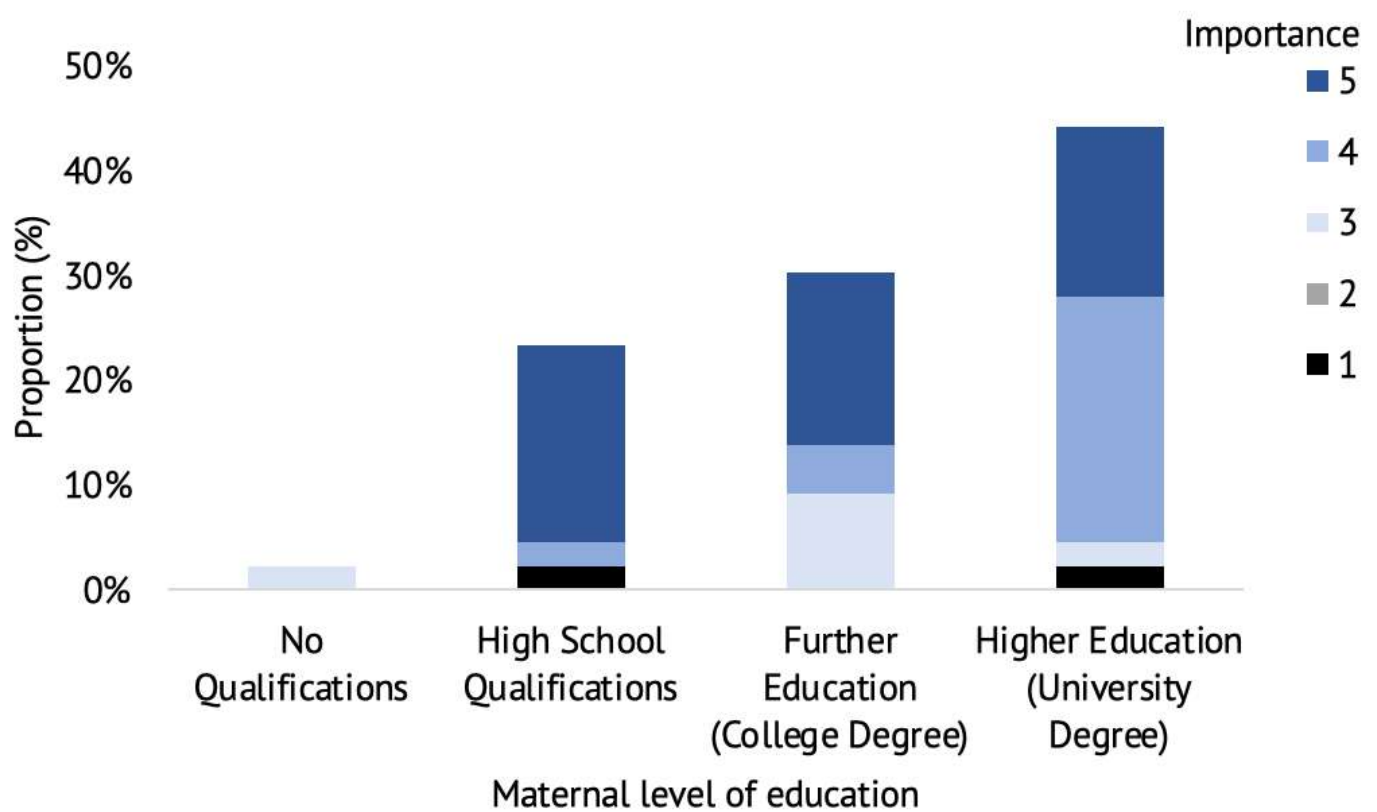

Supplement: sj-pdf-4-jhs-10.1177_17531934211064185 - Supplemental material for The importance of embryology for parents of children with congenital hand differences [file sj-pdf-4-jhs-10.1177_17531934211064185.pdf]

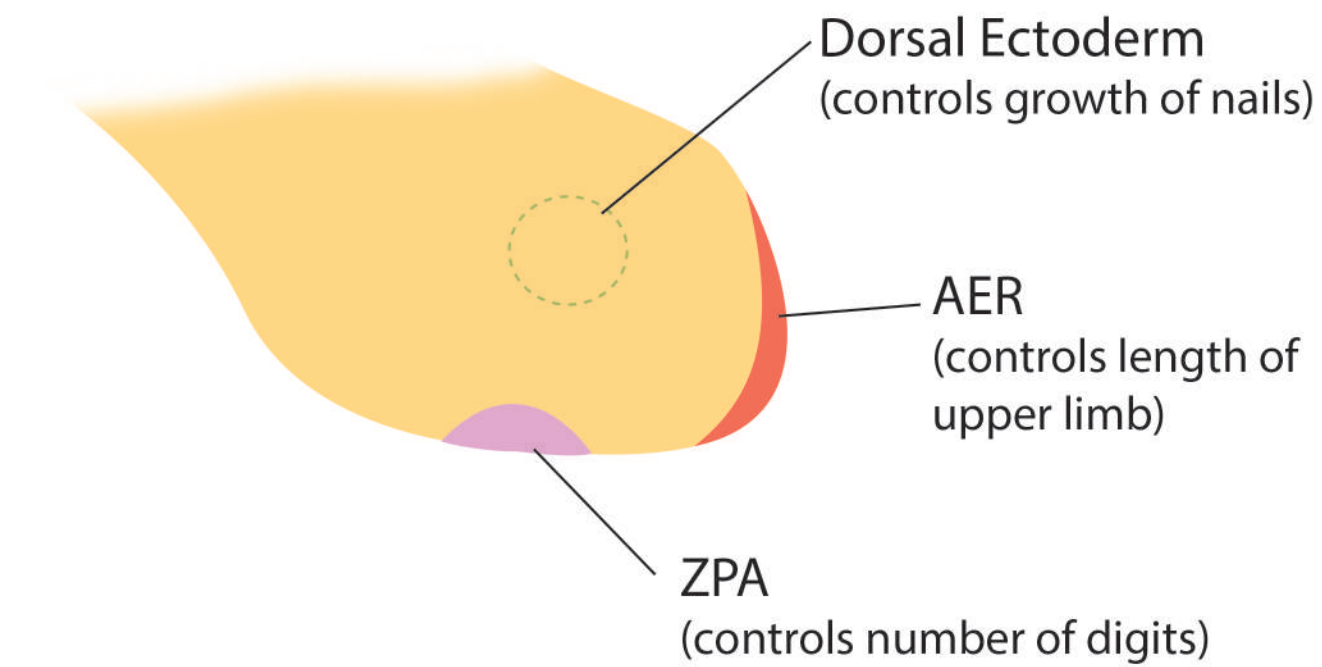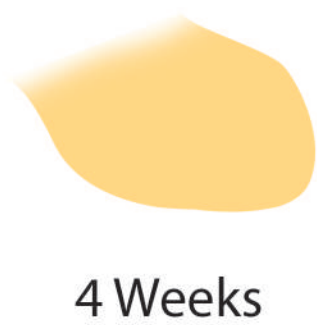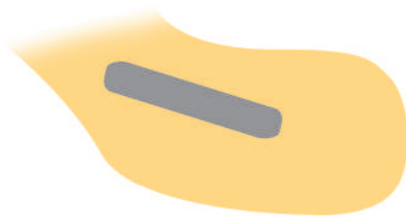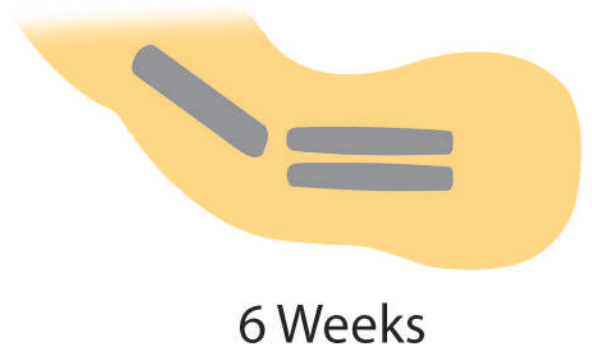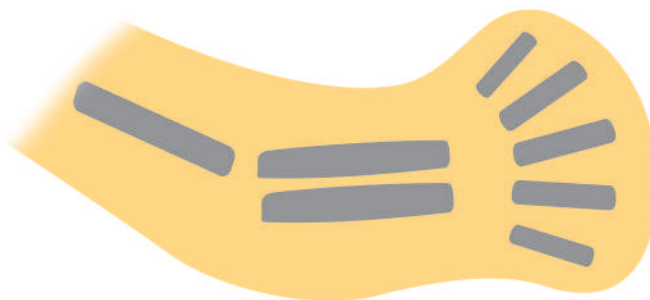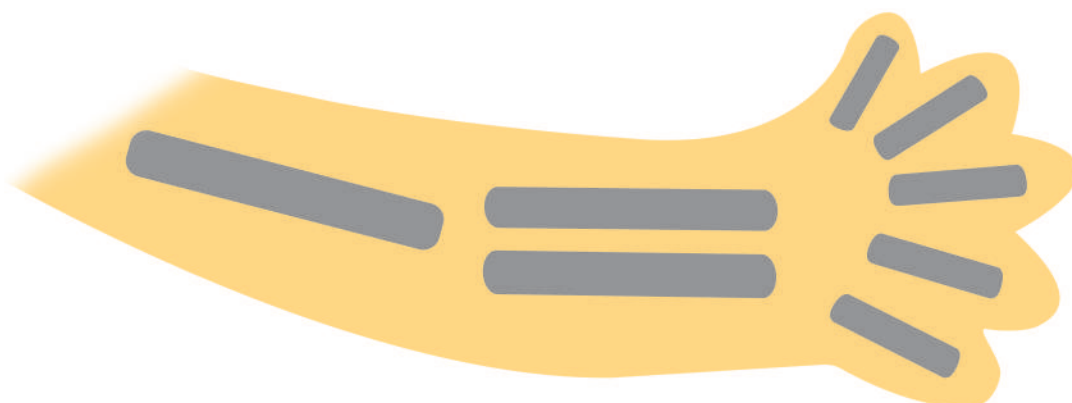

8 Weeks

Supplement: sj-pdf-5-jhs-10.1177_17531934211064185 - Supplemental material for The importance of embryology for parents of children with congenital hand differences [file sj-pdf-5-jhs-10.1177_17531934211064185.pdf]
