# Supplementary material for: The importance of embryology for parents of children with congenital hand differences
Source: J Hand Surg Eur Vol. 2021 Dec 8;47(5):475–80. doi: 10.1177/17531934211064185 (PMC9008554; doi:10.1177/17531934211064185)
Supplement: sj-pdf-6-jhs-10.1177_17531934211064185 - Supplemental material for The importance of embryology for parents of children with congenital hand differences [file sj-pdf-6-jhs-10.1177_17531934211064185.pdf]

Parents' Guide to Understanding the Causes of Congenital Hand Differences (CHDs)

This leaflet aims to provide helpful information for parents and carers of children born with hand differences. It gives a very simple overview of how and why hand differences happen as well as the support that is available. If you would like to discuss treatment options, please speak to the Congenital Hand Team for more information.

What are CHDs?

'Congenital' means 'from birth'. Congenital hand differences (CHDs) occur when a child is born with a hand that had formed differently from what we would expect of a 'normal' hand. This happens in up to 1 in 2,000 live births. Please note that we prefer the term 'differences' rather than 'abnormalities' because that is exactly what it means, i.e., it is just 'different' rather than 'abnormal'.

How do CHDs happen?

Embryology is the study of how a foetus develops. This image shows how the human embryo grows inside the womb. Note that the hand is fully formed within 4 to 8 weeks of a woman becoming pregnant.

The developing embryo uses a network of signals which work together to form the hand. This happens in three directions to produce the 3D shape of the fully developed human hand.

One of the most important regulators of this development is a protein called **sonic hedgehog *(SHH)***. Sometimes, there is either too much or too little *SHH*, which causes the hand to develop differently.

Depending on when and where in the growth pathway these signals differ, development of the upper limb and hand will be affected in different ways.

The lines show how long an embryo is at

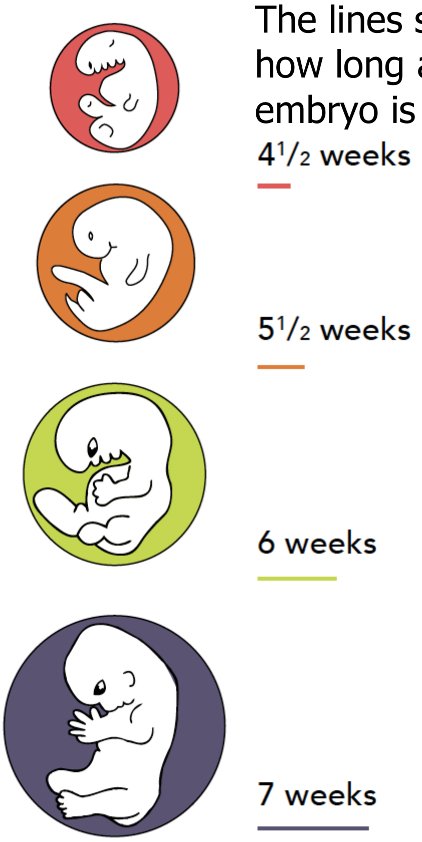

Image with permission courtesy of Reach.

Why has this happened?
It is not yet clear why CHDs happen. They may be due to genes – which 'plan' how the foetus develops in the womb – or the environment in which the baby grows inside the womb, or the external environment.

However, we know that most CHDs happen for unknown reasons and are not related to the genes or behaviours of parents. **It is important to remember that in the overwhelming majority of cases, parents are not to blame when CHDs happen.**

Further support

- You will be able to discuss your child's hand difference when you see a Hand Surgeon who specialises in treating children's hand differences.
- Your clinician may also offer to refer you and your child to a geneticist if you have a family history of limb differences
- Your health board/trust may offer a psychosocial support service for children and families

There are also several support charities for children and families affected by CHDs which we encourage you to consider:

Reach – https://reach.org.uk

- Reach offers families the opportunity to connect and share stories with other families affected by upper limb differences. Through their website and family weekend events, this charity provides useful information for families as a child reaches each milestone

Kidscape – https://www.kidscape.org.uk

- Kidscape teach children proven techniques to build self-confidence

Changing Faces – https://www.changingfaces.org.uk

- Changing Faces offer emotional support, advice and information, seek to develop positive attitudes and better cope with feelings.

Contact
Finally, the Congenital Hand Team are very happy to answer any questions you may have.

Royal Hospital for Sick Children

Authors: AD Clelland, Ó Duncan, WL Lam
